# NF-κB and NLRP3 gene expression changes during warm hepatic ischemia-reperfusion in rats with and without silibinin 

**Published:** 2021

**Authors:** Setareh Zarpou, Hadis Mosavi, Abouzar Bagheri, Majid Malekzadeh Shafaroudi, Abbas Khonakdar-Tarsi

**Affiliations:** 1 *Department of Medical Biochemistry and Genetics, Faculty of Medicine, Mazandaran University of Medical Sciences, Sari, Iran*; 2 *Department of Clinical Biochemistry, School of Medicine, Babol University of Medical Sciences, Babol, Iran *; 3 *Anatomy and Cell Biology Department, Faculty of Medicine* *,* *Mazandaran University of Medical Sciences, Sari, Mazandaran, Iran*; 4 *Faculty of Medicine, Immunogenetic Research Center (IRC), Mazandaran University of Medical Sciences, Sari, Iran *

**Keywords:** Ischemia, NF-Κb, NLRP3, Reperfusion, Silibinin

## Abstract

**Aim:**

This research examined silibinin's anti-inflammatory outcomes on the NOD-like receptor protein-3 (NLRP3) and NF-κB gene expression, which plays a notable role in inciting inflammatory pathways.

**Background:**

Hepatic ischemia-reperfusion (I/R) is a common phenomenon in many clinical cases, including liver surgery and transplantation. Inflammatory mediators are vital contributors to the expansion of hepatic damage after I/R injury (I/RI), and therefore, targeting inflammation is a considerable candidate for the management of hepatic I/RI and its complications.

**Methods:**

Thirty-two male Wistar rats were divided equally into four groups: 1) Control (Vehicle) group, in which rats underwent laparotomy and received normal saline; 2) SILI group, in which rats underwent laparotomy, and 30 mg/kg silibinin was injected intraperitoneal (IP); 3) I/R group, in which rats underwent I/R and received normal saline; and 4) I/R + SILI group, who encountered I/R after laparotomy and received silibinin. After one hour of ischemia and three hours of reperfusion, blood and liver tissue samples were assembled for future biochemical, histological, and gene expression studies.

**Results:**

*In vivo* analysis attested that serum AST and ALT activities were significantly lessened by silibinin in the SILI + I/R group (*p* <0.001). Silibinin ameliorated inflammatory liver tissue injuries, including neutrophil and macrophage infiltration, hepatocyte degeneration, cytoplasmic vacuolation, vascular endothelial damages, and sinusoid dilation observed in the I/R group. During I/R, NLRP3 and NF-κB gene expression showed a significant increment compared to the control group (*p* <0.001), which could be alleviated by silibinin (*p* <0.01).

**Conclusion:**

The results evidence that adjusting the expression of NLRP3 and NF-κB genes during I/R is probably one of the mechanisms of the anti-inflammatory effects of silibinin.

## Introduction

 Hepatic I/R is frequent in various clinical situations, including surgery, biopsy, trauma, and liver transplantation (1). Many previous articles have reported that inflammation, one of the leading causes of hepatic I/RI and its related compilations, conducts a systemic inflammatory response and damages distant organs such as the brain, kidneys, and lungs (2-4). In the acute phase of hepatic I/R, Kupffer cells are activated through damage-associated molecular patterns (DAMPs) released from damaged hepatocytes (5). Activation of Kupffer cells provokes a release of reactive oxygen species (ROS) (mainly from sources of myeloperoxidase) and pro-inflammatory factors, including tumor necrosis factor-α (TNF-α), interferon-gamma (INF-γ), interleukin (IL)-12, and IL-1β (6-8). The large volume of generated ROS is the most important I/R damage mechanism, leading to the peroxidation of membranes, various proteins, and DNA (9). Therefore, accurate knowledge of inflammatory damage mechanisms helps control and reduce I/RI. 

Inflammasomes are cytosolic multi-protein oligomers that can detect microbial pathogens and dangerous endogenous signals of stress or cell damage. Inflammasome complexes are members of the NOD-like receptor protein (NLRP). NLRP3, an NLR protein-containing pyrin's functional domain, is the most prominent and active component of the inflammasome complex (10, 11). The direct binding of DAMPs to the leucine-rich domain of the inflammasome complex disrupts the self-inductance of the complex and causes oligomerization. Their triggering activates caspase-1. Caspase-1, in turn, promotes the maturation of other inflammatory cytokines, ProIL-1β and ProIL-8. These events lead to the start of a series of inflammatory reactions (12-14). Studies have recorded that in addition to monitoring pathogens, inflammasomes are activated in response to endogenous stresses such as ischemia (15, 16). It is known that the inhibition of nuclear factor-kappa B (NF-κB) prevents the overexpression of NLRP3 in inflammatory conditions (14). 

NF-κB is a crucial transcription factor that participates in inflammatory responses during hepatic I/RI (17). This transcription factor exists in the cytoplasm as a heterodimer of p50 and p60, bound to complexes with inhibitory proteins known as the I-kappa-B (I-κB) family. NF-κB has been confirmed to cleave from its inhibitory protein under inflammatory conditions, enter the nucleus, and stimulate the expression of various inflammatory cytokines such as TNF-α and IL-1β (18). Therefore, decreased expression of NF-κB and NLRP3 genes can reduce the inflammatory response intensity during I/R (19-21). In this regard, the efficacy of silibinin in tissue ischemia models has been confirmed in previous reports (22, 23). Studies have shown that silibinin, the principal constituent derived from milk thistle (Silybum Marianum), contributes to several biological functions. It can induce antioxidant enzymes and prevent glutathione depletion, lipid peroxidation, and ROS production under oxidative stress (24-26). As the anti-inflammatory characteristics of silibinin have been proven in various studies (22, 27-29), this inquiry aimed to investigate its effectiveness on the expression of NF-kB and NLRP3 genes during I/R. 

## Methods

**Animals**

In this intervention study, a total of 32 male Wistar rats weighing 250 ± 20 g were procured from the Laboratory Animal Research Center of Mazandaran University of Medical Sciences. One week before initiation of the experiment, the rats were brought to the experimental room to adapt to the environment. They were kept under controlled conditions at 23±2 °C and 55±5 % humidity and a 12-hour dark-light cycle in a ventilated room with free access to water and regular nutrition. Animals were handled according to the experimental methods approved by the Animal Ethics Committee of Mazandaran University of Medical Sciences, Iran, with the approval number (IR.MAZUMS.REC.1398.861).

**Experimental Design**

The rats were randomly divided into four groups, each containing eight rats: 

1) The control or vehicle rats underwent laparotomy only, and their liver was detached from the body to make them look similar to the other groups. Normal saline was injected into this group. 2) In the SILI group, rats underwent laparotomy only and received 30 mg/kg of silibinin (IP). 3) The I/R group were insulted by I/R and received normal saline. 4) The I/R + SILI group accepted I/R after laparotomy and received 30 mg/kg silibinin.

Eighteen hours before surgery, food was removed from the animal cages, but drinking water was freely accessible. Ketamine (60 mg/kg) and xylazine (10 mg/kg) were used intraperitoneally to anesthetize the rats. All surgery protocols were carried out under sterile conditions and between 9-14 o'clock to prevent time variables. 

**Method of causing ischemia**

Through a long incision in the abdomen's midline below the sternum (laparotomy), and after removing the fascia and cutting the rectus abdominal muscle, the liver appeared. Connections between the liver and peritoneal diaphragm were cut, and with a gentle pressure of both hands on the incision sides, the liver was dislodged from the abdominal cavity. A bulldog clamp used to induce one-hour ischemia to the left branches of the triple port arteries, including the hepatic artery, the portal vein, and the bile duct of the left and middle lobes. To avoid intestinal congestion and mesenteric clogging, the right and caudal lobes had a free blood flow. This method caused seventy percent of the ischemia. After 60 minutes of ischemia, the clamp was removed to restore blood flow. Throughout the ischemia, to prevent drying out, a sterile gauze impregnated with normal saline was placed on the liver. During this time, rats were re-anesthetized with ketamine (30 mg/kg) whenever necessary. Following the end of the ischemia period, the clamp was slowly pulled out, the liver was relocated into the abdominal cavity, and the incision site was sutured. Control animals were set up similarly, but no clamps were put on their left and middle lobe vessels.

**Silibinin injection**

Silibinin was purchased as lyophilized with a formulation of silibinin dihydrogen succinate (Cologne, Germany). The high water-soluble silibinin was dissolved in normal saline before injection. It was injected intraperitoneally at a dose of 30 mg/kg twice, each time in a volume of 0.5 mL, before surgery and again immediately after the start of reperfusion.

**Biochemical analysis**

Two-mL samples of blood were taken from the inferior vena cava under general anesthesia using a syringe and kept in sterile glass tubes for half an hour to form a clot. They were then centrifuged at 3000 rpm for 10 minutes. The serum was separated and kept in a 1.5 mL vial at -70 °C until the onset of AST and ALT biochemical analyses. The activities of ALT and AST enzymes were measured by biochemical autoanalyzer (BT-3000-plus, Biotechnica, Italy) using the Pars Azmoon test kit (Iran).

**RNA extraction and real-time PCR**

Small pieces of liver tissue were later stored in RNA (tissue storage reagent) at -70 °C for future evaluation of the gene expression. The total RNA of all liver tissue sections was extracted using an RNeasy plus mini kit (Qiagen, Germany) according to the manufacturer’s guidelines. A UV spectrophotometer (Thermo Scientific, USA) was employed to evaluate RNA concentration at 260 nm, and its purity at an absorbance ratio of 260/280 nm. Moreover, RNA quality was confirmed by two sharp bands detected for 18S and 28S ribosomal RNA by resolving electrophoresis in agarose gel stained with SYBR Green. According to the kit protocol, a concentration of 1 μg of RNA per reaction was applied for the cDNA synthesis (EURx, Poland). Reverse transcription-real time PCR was performed according to the following mixture: 50 ng of cDNA (2 µL), 10 pM of specific primers (1 µL of forward and reverse), 12.5 µL SYBR Green PCR Master Mix reagent (EURx, Poland), and DD water up to 25 µl total volume. The PCR cycles were as follows: UNG pre-treatment at 50 °C for 2 min, initial denaturation at 95 °C for 12 min, and 40 cycles (95 °C for 15 seconds (denaturation), 58 °C for 30 seconds (annealing), and 72 °C for 5 min (final extension)). Glyceraldehyde 3-phosphate dehydrogenase (GAPDH) gene was used for normalization of the results. The sequences of the primers of the NF-κB, NLRP3, and GAPDH are listed in Table 1.

**Table 1:** Sequences of the primers of NF-κB, NLRP3, and GAPDH

Primer sequences (5'→3')	Target genes
Sense: 5' - GACGACACCTCTACACATAGCAG -3' Antisense: 5'- TTCTTCTCCAGCCTTCTCCCA -3'	NF- κB
Sense: 5' - GTCCAGTGTGTTTTCCCAGAC -3' Antisense: 5'- TTGAGAAGAGACCTCGGCAG -3'	NLRP3
Sense: 5'- GAAGGTCGGTGTGAACGGATTTG -3' Antisense: 5'- AATGAAGGGGTCGTTGATGGC -3'	GAPDH

**Tissue collection and preparation**

For pathological examinations, 1-mm pieces of liver tissue were taken from the ischemic lobe and immediately washed in physiological serum to remove any blood. The samples were kept in 10% formalin at room temperature until further investigation. To prepare the tissues and remove fixatives, they were first washed with water, and dehydration was then performed with different alcohol concentrations (100-50%). The samples were then cleaned with xylene and finally placed in paraffin. Sections of 3–5 μm of liver tissues were cut with a microtome, stained into hematoxylin and eosin (H&E) dye, and finally viewed under a light microscope to assess the degree of damage. 

**Statistical analysis**

Data was analyzed using SPSS 18 software. The results of real-time PCR were analyzed using Linreg and Rest-reg software. All results were described as mean ± standard deviation (SD). Differences between the groups were determined by one-way ANOVA and Tukey multiple comparison in SPSS software, and the significance level was considered to be *p* <0.05.

## Results

***NF-***κ***B and NLRP3 gene expression***

**Figure 1:**
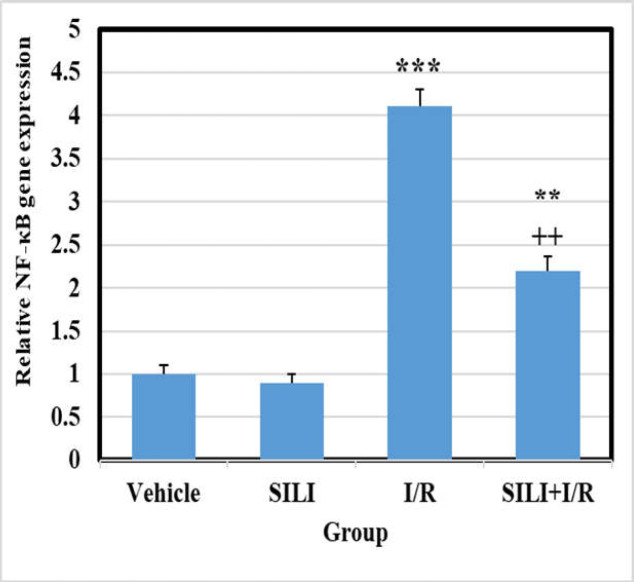
Silibinin attenuates the mRNA expression of NF-κB in liver tissue after hepatic I/R. Results are shown as mean   standard deviation (mean SD) with eight rats in each group. ^*** ^*P* 0.001 indicates a significant difference compared to the control group, and ^++ ^*P* 0.01 indicates a significant difference compared to the I/R group. NF-κb: Nuclear factor-kappa B; SILI: silibinin; I/R: ischemia/reperfusion

**Figure 2:**
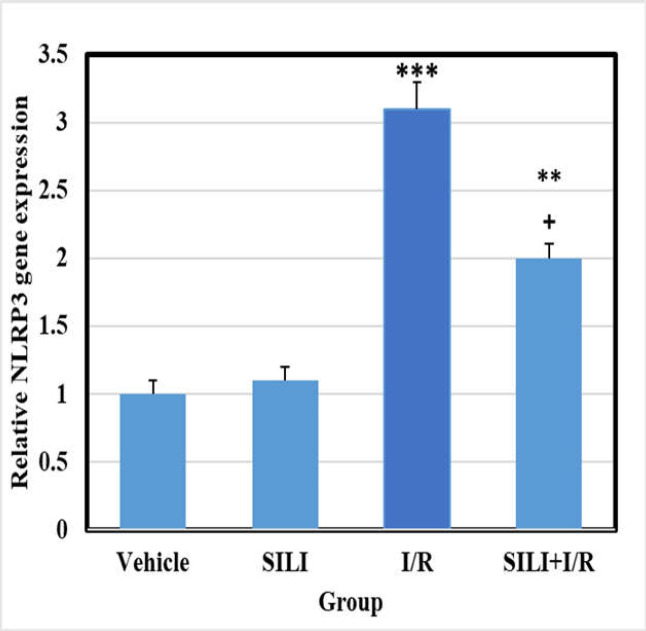
Silibinin reduces the mRNA expression of NLRP3 in liver tissue after hepatic I/R. All results were shown as mean   standard deviation (mean SD) with eight rats in each group. ^*** ^*P* 0.001 indicates a significant difference compared to the control group, and ^+ ^*P*  0.05 indicates a significant difference compared to the I/R group. SILI: silibinin; I/R: ischemia/reperfusion; NLRP3: NOD-like receptor protein

Analysis of real-time PCR results showed that NF-κB and NLRP3 mRNA levels were not markedly different between the vehicle and SILI groups. However, in the I/R group, the amount of NF-κB and NLRP3 gene mRNA increased significantly compared to the vehicle group (*p *0.001). Treatment with silibinin in the I/R+SILI group could significantly reduce the expression of the genes compared to the control group, although it could not hit the level of the control group (Figures 1 and 2) (*p *0.01 and *p *0.05).

***Serum ***activities ***of liver enzymes***

**Figure 3:**
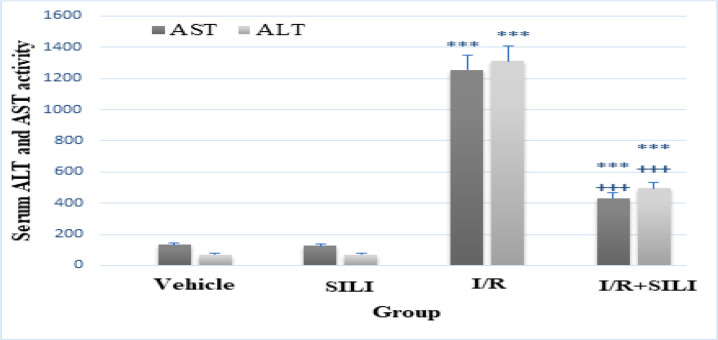
Silibinin attenuates the levels of ALT and AST in serum during hepatic I/R. All data are shown as mean   standard deviation (mean SD) with eight rats in each group. ^*** ^*P* 0.001 indicates a significant difference compared to the control group, and ^+++^ *P* 0.001 indicates a significant difference compared to the I/R group. SILI: silibinin; I/R: ischemia/reperfusion; AST: aspartate aminotransferase; ALT: alanine aminotransferase

As shown in Figure 3, there were no notable differences in activities of liver AST and ALT enzymes between the control and SILI groups (*p* ˃0.05). In the I/R group, AST and ALT activities increased significantly compared to the control group (*p* <0.001). Silibinin treatment could significantly reduce this increase in the I/R+SILI compared to the I/R group (*p* <0.001).

***Liver histopathology***

**Figure 4:**
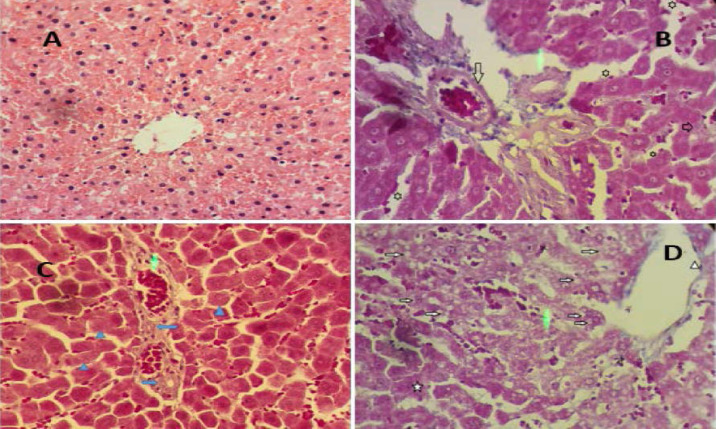
Evaluation of rat liver histology in four groups (magnification 400 x). A) Vehicle group, B) SILI group, C) I/R group, and D) I/R+ SILI group. SILI: silibinin, I/R: ischemia-reperfusion

Figure 4A shows that the lobular central vein; its endothelial cells are intact. Hepatocytes are healthy and have a definite membrane boundary. The cytoplasm of hepatocytes is pink to red, and most of its organelles are mitochondria (2500-3000 mitochondria per hepatocyte). 

According to the findings in Figure 4B, the hepatic classical lobule tissue is seen completely healthy in different zones. In the portal space, intact blood vessels, their branches, and bile ducts are seen. In the portal space and the surrounding classical zone I, liver cords with high stainability, the healthy nuclei with apparent nucleoli, and a small number of apoptotic vacuoles indicate the hepatic tissue is healthy. In this group, normal sinusoidal space with intact endothelial lining and a large number of Kupffer cells are also observed. Surrounding portal space, bile ducts, portal vein branches, and mitochondrial density are normal in I and III zones of the classical hepatic lobules. However, multiple apoptotic vacuoles with different sizes, colorless nuclear chromatin, and lower cytoplasmic stainability are characteristic of mitochondrial fusion and destruction in the portal space following ischemia. The endothelial cells of the sinusoidal wall have desquamated in some zones, but the endothelium covering the portal vein branches and the simple cubic epithelium covering the bile duct and the cholangioles are intact. Additionally, in the endothelium lining the lobule central vein appears intact (Figure 4C). 

In the I/R+ SILI group, in zone III of the hepatic lobule, the hepatic cords of the central vein are damaged. Hepatocyte nuclei often lost their nucleoli, and the intensity of nucleus chromatin stainability was reduced. Also, bubbles or apoptotic vacuoles accumulated in the cell cytoplasm that joined together to form a signet ring cell (SRC). SRC is a pathological form of ischemia or liver cirrhosis. In ischemic hepatic sections treated with silibinin, the number of SRCs in zone III of the classic hepatic lobule was limited. In general, it can be concluded that in tissue sections that were affected by silibinin, the liver tissue damage was reduced in comparison with ischemic tissue sections, especially in zones I and III of the classic hepatic lobules (Figure 4D).

## Discussion

Hepatic I/RI mainly occurs in various clinical circumstances, including liver transplantation, and is associated with liver dysfunction and mortality (1). Accumulating investigations have confirmed that inflammatory mechanisms participate in the pathophysiology of hepatic impairment subsequent I/R injury, and therefore inhibiting immune responses and inflammation is beneficial (30, 31). The current experimental study found that 30 mg/kg silibinin injection improved liver tissue damage during I/R by reducing NLRP3 and NF-κB gene expression.

Hepatic I/R is a pathophysiological process, triggering various molecular mechanisms that provoke further hepatocellular damages or even death (32, 33). Studies performed in animal models have identified many of the mechanisms involved in the pathophysiology of liver I/R injury and have reported that inflammasome activation in liver tissue is critical in liver I/R injury (15). NLRP3 makes a fundamental contribution to generating inflammatory responses through secreting a series of pro-inflammatory cytokines that might cause endothelial dysfunction, leukocyte infiltration, and, ultimately, hepatocellular damage during I/R (34-36). Furthermore, it is well known that NF-κB signaling activation is involved in accelerating inflammatory responses and pathogenesis of hepatic I/R injury by inducing pro-inflammatory cytokine production (17, 37). These inflammatory processes are known to promote neutrophil production, chemokines attraction, and vascular cell adhesion molecules. The events result in the adhesion and infiltration of neutrophils from the vessel lumen into the hepatic parenchyma, ultimately leading to liver damage and dysfunction (34, 38, 39). Therefore, many studies have summarized that the inhibition of NF-κB and NLRP3-dependent inflammation is a practical approach to protecting the liver and other organs against I/R injury in pre-clinical animal models (19). Recently, He et al. reported resveratrol ameliorates cerebral I/R injury through NLRP3 inflammasome inhibition in a rat model (40). In another comparison, Wang et al. reported that curcumin, a polyphenolic compound derived from *Curcuma longa*, attenuated hepatic I/R injury by inhibiting the TLR4/NF-κB pathway (17). Lingappan et al. demonstrated that during I/R, NF-κB activation caused an elevation in the transcription of IL-12, IL-1β, and TNFα through the signaling pathway of DAMPs (41, 42). Kim et al. showed that in HMC-1 human mast cells, inflammatory responses could be repressed by silibinin within the impeding of the NF-κB signaling pathway (43). Silybin suppresses NLRP3 inflammasome stimulation and NF-κB signaling in mice and results in attenuating acute LPS-induced lung damage (44). In vitro investigation of the NLRP1/NLRP3 inflammasomes and p65NF-κB activity showed they were decreased by silibinin in monocytes from preeclamptic women (45). Therefore, there is a strong correlation between NLRP3 and NF-κB activity and inflammatory responses. 

Overall, based on the current data, hepatic I/RI is successfully verified in a rat model. This experiment determined that silibinin could suppress hepatic I/R-induced inflammatory responses by decreasing the mRNA expression of NF-κB and NLRP3 in rats. It was also revealed that silibinin suppressed the liver biomarkers' serum levels, indicating its capacity to attenuate hepatic damage in I/R. The histological study in this investigation showed that hepatic tissue was damaged in I/R rats, in line with the results of molecular experiments obtained from the present study. The findings of this study were similar to the results of other studies. Kyriakopoulos et al. found that silibinin improved renal parenchyma in rat kidneys after hepatic I/R (46). Using an I/R rat model, Kordkheyli et al. showed that silibinin inhibited endothelium damages, hepatocyte vacuolization, and sinusoidal congestion during hepatic I/R damage (22). Among the important results obtained is that nuclei lose their nucleoli and their chromatin stainability in the I/R groups in hepatocytes. Moreover, apoptotic vacuoles congested in the cytoplasm and formed SRCs, a pathological form of hepatic ischemia. Silibinin could decrease SRCs in zone III of the classic hepatic lobule and ameliorate hepatic tissue damage during I/R.

The data showed that silibinin suppressed hepatic I/R injury, possibly by inhibiting NF-κB and NLRP3 mRNA expression along with other effects presented previously. Silibinin also attenuated the serum activities of liver enzymes, demonstrating its capability to suppress hepatic damage during I/R.
